# Traditional Chinese Medicine and Autonomic Disorders

**DOI:** 10.1155/2015/429181

**Published:** 2015-07-29

**Authors:** Kazuo Toda, Jorge L. Zeredo, Sae Uchida, Vitaly Napadow

**Affiliations:** ^1^Integrative Sensory Physiology, Graduate School of Biomedical Sciences, Nagasaki University, Nagasaki 8528588, Japan; ^2^Graduate Program in Health Sciences, University of Brasilia, 70910-900 Brasilia, DF, Brazil; ^3^Department of the Autonomic Nervous System, Tokyo Metropolitan Institute of Gerontology, Itabashi, Tokyo 1730015, Japan; ^4^Athinoula A. Martinos Center for Biomedical Imaging, Massachusetts General Hospital, Harvard Medical School, Charlestown, MA 02129, USA

Traditional Chinese Medicine (TCM) consists of acupuncture, moxibustion, qigong, and herbal medicine. In modern times, TCM has often been used as complementary treatment for diseases that do not respond satisfactorily to conventional medicine. Autonomic disorders, which are caused by chronic imbalance of vegetative organs as well as autonomic-related areas in the central nervous system, fall into this category of difficult-to-treat conditions. Therefore, a significant role for TCM has been increasingly accepted in both Asian and Western countries for the treatment of autonomic disorders. The purpose of this special issue was to gather the most up-to-date evidence concerning the effective use of TCM for the treatment of autonomic disorders. Particular focus in this issue was placed on the effective use of herbal medicine.

Three papers are related to digestive function. Inflammatory bowel disease (IBD) is a chronic intestinal disorder which is characterized by bouts of severe intestinal inflammation and colonic mucosal ulcer. D. Liu et al. showed that Si Shen Wan significantly inhibited apoptosis of IECs (intestinal epithelial cells) through activation of PLC-*γ*1 and suppression of P13K/Akt signal pathway. Their results indicate that Si Shen Wan (which consist of four components) can effectively treat colitis by inhibiting excessive apoptosis of IECs.

A basic study on the effects of Huqi San (Qi-protecting powder) for cholestasis was conducted by X. Xue et al. They investigated the positive effects of Huqi San in the rat, indicating that it can stimulate Cl ion secretion in the distal colon by means of increasing the mRNA transcript and protein content of the cystic fibrosis transmembrane regulator in the liver, distal colon, and pancreas. They suggest that transporter modulation at a transcriptional level may serve as a potential target for a new therapeutic strategy for cholestasis.

Swertianlarin, isolated from* Swertia mussotii* Franch. and* Enicostemma axillare*, has hepatoprotective effects against cholestasis. In China, it is often used for treatment of jaundice. A pilot study was conducted by L. Zhang et al. using a rat bile duct-ligation (BDL) model. Serum bile acid levels were reduced in BDL rats treated for 14 days with Swertianlarin. This observation suggests that* Swertia mussotii* Franch. attenuates liver injury, inflammation, and cholestasis. Further studies are critically needed on whether Swertianlarin exerts the protective role in other cholestatic models and what the molecular mechanism underlying Swertianlarin-induced protective effects on liver injury, inflammation, and cholestasis is.

Hyperglycemia and diabetes-related depression can be improved by Chinese herbal medicine, Zuogui jiangtang jieyu (ZGJTJY) in rats. Y. Wang et al. conducted an interesting study using a 28-day mild stress model together with consistent high-fat diet and injection of streptozotocin. They investigated whether ZGJTJY prescription has hypoglycemic and antidepressant effects and found that ZGJTJY inhibits the expression of 11*β*-hydroxysteroid dehydrogenase type 1 (11*β*-HSD1) and increases glucocorticoid (GR) levels in the hippocampus and subsequently modulates blood glucose levels. They concluded that this potential property of ZGJTJY could be of benefit for the treatment of behavior and cognitive function in diabetes-related depression.

Shenfu Decoction (SFD) is one of the most popular herbal medicine formulas with a long history of use in China. This herbal medicine consists of a mixture of* Panax ginseng* and Radix Aconiti Lateralis Preparata. Decoction is a typical traditional method of extraction by boiling of dissolved chemicals from herbal material and widely conducted in China, Japan, Korea, and other Asian countries. A study by J. He et al. had the main purpose of developing a rapid and sensitive Ultra Performance Liquid Chromatography/Quadrupole Time-of-flight Mass Spectrometry method. This technique is thought to be important to analyze pharmacokinetics of multiple components in various herbal medicines.

We believe that this special issue will provide many readers with relevant up-to-date information in the fields of autonomic nervous system and traditional medicines.

*Kazuo Toda* *Kazuo Toda* *Jorge L. Zeredo* *Jorge L. Zeredo* *Sae Uchida* *Sae Uchida* *Vitaly Napadow* *Vitaly Napadow*

